# Long-term effects of combined mindfulness intervention and app intervention compared to single interventions during the COVID-19 pandemic: a randomized controlled trial

**DOI:** 10.3389/fpsyg.2024.1355757

**Published:** 2024-03-19

**Authors:** Constance Karing

**Affiliations:** Department of Research Synthesis, Intervention and Evaluation, Institute of Psychology, Friedrich-Schiller-University, Jena, Germany

**Keywords:** mindfulness intervention, active control, students, a mindfulness app, 7Mind, follow-up, RCT

## Abstract

**Objectives:**

The study examines the short-, middle-, and long-term effects of a combined intervention (face-to-face mindfulness intervention plus the mindfulness app 7Mind), compared to single interventions (face-to-face mindfulness intervention alone and an intervention via app 7Mind alone). The subgroups were compared with an active control group on mindfulness, mindful characteristics, mental health, emotion regulation, and attentional abilities during the COVID-19 pandemic. Additionally, the study explores whether students’ engagement with the app and their formal mindfulness practice at home improves intervention outcomes.

**Methods:**

The study employs a randomized controlled trial approach involving three intervention groups and an active control group, with two follow-ups conducted over 12 months. The study included 177 university students who were randomly assigned to a mindfulness group (*n* = 42), a mindfulness app group (7Mind app, *n* = 44), a mindfulness + app group (*n* = 45), and an active control group (*n* = 46). The duration of the interventions was 4 weeks. The outcome variables were assessed at pre- and post-intervention, at 4 and 12 months post-intervention.

**Results:**

At post-intervention and during both follow-ups, students in the combined mindfulness intervention did not demonstrate better outcomes compared to students in the single interventions or the active control group across all measures. Additionally, no statistically significant difference was observed between all interventions and the active control groups on any of the measures. However, it is noteworthy that all intervention groups and the active control group exhibited improvement in mindfulness, body awareness, emotion regulation, stress, and attentional abilities over the short, medium, and long term. Moreover, higher app usage in the app groups was significantly associated with increased body awareness. However, greater app use was also correlated with higher stress.

**Conclusion:**

The results suggest that the mindfulness intervention and the mindfulness app were similar to the active control condition (communication training) on the investigated variables in the short, medium, and long term. Furthermore, an increased use of a mindfulness app can negatively affect stress.

## 1 Introduction

Even before the COVID-19 pandemic, mental health problems were prevalent among university students (e.g., Auerbach et al., 2018) and were associated with poor academic performance, increased dropout risk, lower study satisfaction, as well as impaired relationships and social life (Alonso et al., 2018; Bruffaerts et al., 2018). However, the outbreak of the COVID-19 pandemic and the imposed restrictive measures (e.g., university closures and physical distancing) have caused negative impacts worldwide on students’ life, education, and financial situation (e.g., Aucejo et al., 2020; Appleby et al., 2022). Germany’s first national lockdown was between March and May 2020, followed by a “hard lockdown” from mid-December 2020 until March 2021 (Bundesgesundheitsministerium, 2021). During these lockdowns, universities and other campus facilities remained closed. Furthermore, the broader spread of the COVID-19 virus in the autumn of 2021 forced nearly all universities in Germany to switch to remote learning (Hochschulrektorenkonferenz, 2022). Many students were confronted with changes in teaching methods (e.g., remote learning), social distancing, uncertainties about the future academic situation, and unexpected financial demands (e.g., Khan-Burki, 2020; Lörz et al., 2020).

Several studies have explored the impact of the pandemic on students’ mental health (e.g., Elmer et al., 2020; Conceição et al., 2021; Karing, 2021; Kohls et al., 2023). Elmer et al. (2020) and Conceição et al. (2021) reported that the prevalence of anxiety and depression among students from Switzerland and Portugal increased during the early phase of the COVID-19 pandemic as compared to before the pandemic. Furthermore, Kohls et al. (2023) found in cross-sectional studies that students reported higher levels of depression in 2021 than in 2020. These trends in mental health justify the application of evidence-based psychological interventions addressing mental health issues and needs among university students.

Mindfulness interventions have been applied to university students (e.g., Halladay et al., 2019; Johnson et al., 2023). These interventions aim to foster attention and awareness of one’s present experiences without any judgment (Baer et al., 2008) and have been carried out face-to-face or online through mobile mindfulness apps (e.g., Headspace, Flett et al., 2019) or mindfulness audio recordings (e.g., Devillers-Réolon et al., 2022; Karing, 2023). Several reviews and meta-analyses have shown that face-to-face and online mindfulness interventions can promote mindfulness and mental health and reduce stress in university students even before the COVID-19 pandemic (McConville et al., 2016; Halladay et al., 2019). Follow-up maintenance has been shown only by McConville et al. (2016) for students’ anxiety (1-month follow-up). However, the benefits of online and face-to-face mindfulness interventions on mental health and stress were not found when mindfulness interventions were compared to active control groups at post-test and at short and medium follow-ups (2- to 8-week follow-ups, -; 4- to 20-week follow-ups, Halladay et al., 2019). Similar results were found in a meta-analysis by Dawson et al. (2020). In addition, they found that mindfulness-based interventions did not outperform active control groups for mindfulness and emotion regulation. However, this meta-analysis demonstrated that mindfulness interventions outperformed active controls for stress and state anxiety at post-intervention. Although Johnson et al. (2023) showed in their meta-analysis of pre-pandemic mindfulness intervention studies compared with active control groups that mindfulness training did not improve anxiety, depression, mindfulness, and mechanisms (e.g., emotion regulation and attention control), they reported that the effect sizes trended in favor of the mindfulness interventions. Furthermore, most mindfulness interventions incorporated formal home practice because it is assumed that home practice is critical to changes in intervention outcomes (e.g., Parsons et al., 2017; Barceló-Soler et al., 2023). Parsons et al. (2017) examined the importance of formal home practice in mindfulness-based interventions in their meta-analysis. The authors reported that participants’ formal mindfulness practice at home was positively associated with intervention outcomes (e.g., mental health), where participants practiced the standard amount of home practice (i.e., 270 min per week).

Although previous studies have shown that mindfulness is related to body awareness and non-attachment, and both seem to be mediators in the relationship between mindfulness and mental health (Burzler et al., 2019; Karing et al., 2021), only a few studies have investigated the effect of mindfulness training on these constructs (Beshai et al., 2020; Karing and Beelmann, 2021). Non-attachment is described as a flexible, balanced way of relating to one’s experiences and not cling to or avoiding such experiences (Sahdra et al., 2015). Body awareness is the ability to recognize internal body sensations (Price and Mehling, 2016). Beshai et al. (2020) reported that a 4-week mindfulness intervention with adults resulted in greater non-attachment in the intervention group than in the active control group post-intervention (watched nature videos with meditation music), whereas Karing and Beelmann (2021) showed only a significant effect of a 6-week mindfulness intervention with university students on non-attachment in the per-protocol sample at post-test and at 2.5-months follow-up. For body awareness, we found a greater increase in the mindfulness group compared to an inactive control group at post-test and follow-up (Karing and Beelmann, 2021).

Various mindfulness intervention studies with student samples have been conducted during the COVID-19 pandemic (e.g., Sun et al., 2022; Witarto et al., 2022; Lahtinen et al., 2023). However, face-to-face interventions were reduced during the pandemic because of lockdown and social distancing measures (Witarto et al., 2022). Therefore, many interventions were delivered online (Witarto et al., 2022). Easy access to interventions, continuous availability, greater anonymity, and lower costs are advantages of online interventions (Bossi et al., 2022). Most of these intervention studies investigated only the effects on mental health and mindfulness and only over a short period (e.g., Sun et al., 2022; Lahtinen et al., 2023). Furthermore, mixed results were reported across these studies. A meta-analysis by Witarto et al. (2022) that included eight studies with students, the general population, or workers reported positive effects of online mindfulness interventions on mental health outcomes (e.g., depression, anxiety, and stress) post-intervention during the pandemic. However, only small effects were found for depression and anxiety at follow-up, but no significant effects were observed for stress. In addition, the authors reported that the effect size for anxiety was significantly larger for inactive controls than for active control groups. For depression, there was no significant difference between the effect size of inactive and active controls. Furthermore, Lahtinen et al. (2023) showed in an intervention study with university students and staff that using a mindfulness app for 4 weeks during the 2020 COVID-19 pandemic resulted in higher improvements in stress and depression in the intervention group than in the active controls (psychoeducation online program) at post-intervention. At a 2-month follow-up, there was no significant difference between the intervention and active controls in stress and depression. Moreover, both groups improved in anxiety and mindfulness over the intervention period. In addition, Lahtinen et al. (2023) showed that greater mindfulness app use was related to a higher increase in mindfulness over intervention time. A different finding was reported by Sun et al. (2022). A mindfulness mobile intervention resulted in a greater improvement in anxiety for the intervention group (*d* = 1.40) than for the active controls (social support mobile intervention, *d* = 0.68). In contrast, in both groups, depression decreased, and mindfulness increased at the 2-month follow-up. Similarly, Devillers-Réolon et al. (2022) investigated the efficacy of a brief online mindfulness training on students’ mental health and cognitive difficulties (attentional abilities) during the second French lockdown in the Winter of 2020. The authors found significant short-term effects on depression, anxiety, stress, and wellbeing compared to inactive controls. However, there was no significant effect on students’ attentional abilities.

So far, there has been little research on the efficacy of blended mindfulness interventions (e.g., face-to-face intervention with app use), although it is assumed that blended interventions strengthen the effects of mindfulness trainings and bring these interventions into daily lives (Wentzel et al., 2016; Borjalilu et al., 2019). Furthermore, the repetition of behavior in a consistent context (e.g., home practice) is considered to be important for behavior changes (Lally and Gardner, 2013; Parsons et al., 2017). One study (Borjalilu et al., 2019) reported that a blended mindfulness intervention with university students resulted in greater improvements in depression, anxiety, and stress than interventions solely based on face-to-face sessions or the use of an app at post-intervention.

Therefore, the current study aims to:

1. Investigate the short-, middle-, and long-term effects of a combined intervention that included a face-to-face mindfulness intervention plus the mindfulness app 7Mind, a face-to-face mindfulness intervention alone, and intervention via app 7Mind alone, compared with an active control group on mindfulness, mindful characteristics, mental health, emotion regulation, and attentional abilities during the COVID-19 pandemic.
2. Examine whether students’ use of the app 7Mind improves the change in intervention outcomes.
3. In addition, because participants of the face-to-face mindfulness interventions were encouraged to practice the mindfulness exercises at home, the study aims to investigate whether participants’ formal mindfulness home practice improves the change in outcomes.

It was hypothesized that all intervention groups would have more significant improvements from pre- to post-intervention and both follow-ups in mindfulness, mindful characteristics (body awareness, non-attachment), emotion regulation (reappraisal, acceptance, rumination), mental health (depression, anxiety, stress), and attentional abilities than the active control group. Furthermore, it was expected that the mindfulness intervention plus app would have higher improvements from pre- to post-intervention and both follow-ups on all measures than the single interventions because the app use between face-to-face sessions could help students to consolidate their skills learned in the face-to-face sessions. Lastly, it was hypothesized that students who use the app 7Mind and/or formal mindfulness home practice will demonstrate positive changes in all outcome measures from pre- to post-intervention and both follow-ups.

## 2 Methods

### 2.1 Study design and procedures

The study used a randomized controlled trial approach with three intervention groups and an active control group and with two follow-ups over 12 months. The study received approval from the Institutional Ethics Commission of the University of Jena, Germany (Approval number: FSV 20/043), and written informed consent was obtained from all participants. The interventions received by the three intervention groups were face-to-face mindfulness intervention (mindfulness group), mindfulness intervention via app 7Mind (app group), or face-to-face mindfulness intervention plus mindfulness app 7Mind (mindfulness+app group). The active control group received a face-to-face communication course. The courses were conducted during the COVID-19 pandemic between November 2020 and December 2021. Due to the lockdowns in Germany, the duration of the interventions was reduced to four instead of seven sessions. Booster sessions were offered to all participants after 4 months. However, only 30 (16.9%) participants attended the booster sessions. The interventions were conducted at a university campus.

A prior sample size analysis for repeated measures analysis of variance was conducted using G*Power 3.1 (Faul et al., 2007). The analysis was calculated with four groups and four measurements, a power of 0.80, a small effect size (*f* = 0.10), and α = 0.05. The results indicated a total sample size of 160 students. Accounting for dropout rates found in previous mindfulness intervention studies (Noone and Hogan, 2018; Karing and Beelmann, 2021; Karing, 2023), a sample size of *N* = 200 students should be recruited.

Participants were recruited through flyers distributed at universities, university mailing lists, and social media. The intervention study was advertised as a stress prevention training. Thus, participants were partially blinded to the purpose of the interventions because all participants were informed only about the outcome stress prevention. Assessments (online questionnaires) were conducted immediately before the interventions (pre-intervention, T1), after the interventions (post-intervention, T2), and 4 months (first follow-up, T3) and 12 months after the interventions (second follow-up, T4).

### 2.2 Participants

**Figure 1:**
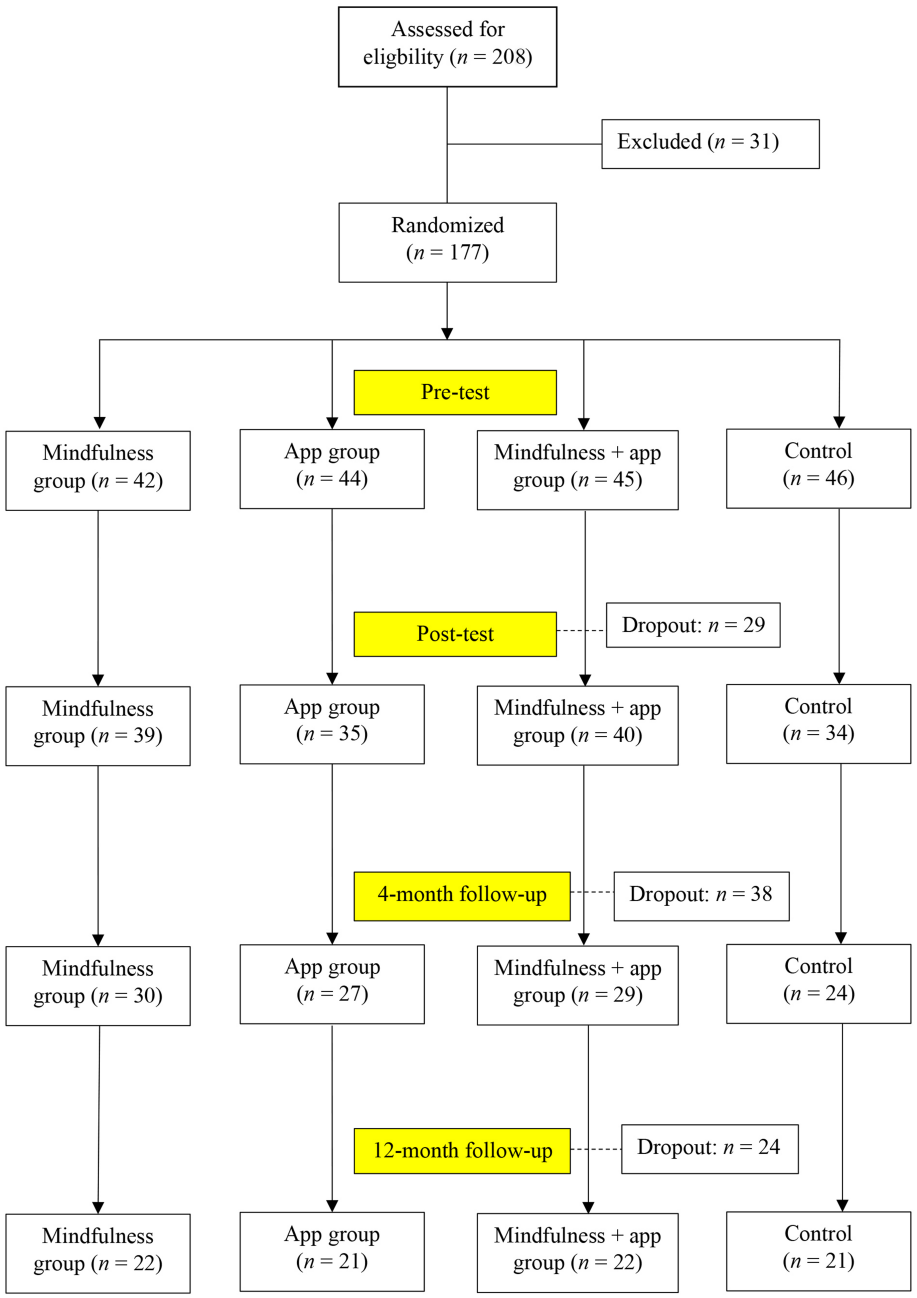
Recruitment and participant flow chart.

A total of 208 university students were recruited for the intervention study. Inclusion criteria included (1) age ≥ 18 years, (2) ability to understand the German language, and (3) their willingness to take part in an intervention study and to provide informed consent. Thirty-one students dropped out before the intervention study started. The main reasons for dropping out were lack of time and illness. Thus, the remaining 177 university students were randomly assigned to the mindfulness group (*n* = 42), app group (*n* = 44), mindfulness + app group (*n* = 45), and active control group (*n* = 46). A total of 148 participants completed the post-assessment questionnaire (T2), 110 responded to the first follow-up questionnaire (T3), and 86 completed the second follow-up questionnaire (T4). Figure 1 shows the flow of participants.

The final sample (*n* = 177) consisted of 145 women (81.9%), 31 men (17.5%), and one non-binary participant. The students’ average age was 22.96 years (*SD* = 4.66). The average academic semester was 3.58 semesters (*SD* = 3.15). Students’ demographic characteristics by group are displayed in Table 1.

**Table 1:** Demographic characteristics at T1 by group.

Variable	Mindfulness + app (*n* = 45)	Mindfulness (*n* = 42)	App (*n* = 44)	Control (*n* = 46)
Age, mean (*SD*)	23.89 (6.96)	22.98 (4.21)	21.93 (2.37)	23.02 (3.75)
Gender, *n* (%, female participant)	33 (73.3)	34 (81.0)	39 (88.6)	39 (84.8)
Semester, mean (*SD*)	3.91 (3.35)	3.19 (3.16)	3.41 (3.20)	3.78 (2.94)

### 2.3 Interventions

#### 2.3.1 Mindfulness app intervention

A commercially available mindfulness app, 7Mind, was used for the mindfulness app intervention. The app 7Mind provides guided mindfulness meditations through mobile applications. After providing informed consent and completing the baseline online survey (T1), the app group met a research member face-to-face. The students were briefed about the intervention study and the app 7Mind in this session. The participants were asked to install the app 7Mind and use a study-specific log-in ID. The participants were instructed to use 7Mind’s free-of-charge basic course during the 4-week study period. The basic course included seven sessions, with each session lasting 7 min. The participants were instructed to complete two mindfulness sessions a week (14 min per week) as defined by the researcher. The exercises included, for example, a body scan, awareness of breathing, awareness of the body, and observing thoughts. After 4 months, the participants were asked via email to use another two mindfulness sessions (booster sessions: forest meditation and global connectedness). Both sessions were free and lasted 10 min.

#### 2.3.2 Face-to-face mindfulness interventions

The mindfulness group and the mindfulness+app group received a 4-week mindfulness training. The intervention included a 90 min session (week 1) and three 30 min face-to-face sessions by a trainer. The interval between the sessions was 1 week. Thus, the students met their trainer once a week. The trainings were performed under precautions such as wearing masks, limiting the numbers of participants per group, and physical distancing. The training was based on our group-based manualized mindfulness intervention, which was positively evaluated in a randomized controlled intervention study among university students (Karing and Beelmann, 2021).

The mindfulness intervention content for both groups was identical. The intervention included awareness of breathing and body scan meditation (week 1), awareness of breathing and body, and observing thoughts (week 2), mindful eating (week 3), and mindful hatha yoga (week 4). Each session included a theoretical introduction to the new mindfulness exercise and the practice and discussion of the new technique. The introduction and practice of each new technique lasted approximately 15 min. The trainer encouraged all participants to practice the mindfulness exercise at home at least once a week.

Correspondingly, the mindfulness+app group was asked to install the app 7Mind and use the app between sessions during the 4-week study period. Similar to the app group, the students were instructed to use 7Mind’s basic course and complete two weekly mindfulness sessions.

Two booster mindfulness sessions were offered to both groups 4 months after the initial mindfulness intervention. The intervention involved a 90 min (week 1) and a 30 min (week 2) face-to-face session with their previous trainer. The first session included a short review of the mindfulness components, an exchange of experience over the last 4 months, and two exercises (awareness of breathing, a new exercise “mindfulness hearing”). The second session included walking meditation as a new exercise.

#### 2.3.3 Active control group

The active control group received a face-to-face communication course during the 4-week study period. The program consisted of 90 min (week 1) and three 30 min (week 2) sessions with a trainer. The interval between the sessions was 1 week. The sessions included an introduction to communication (week 1), communication models (e.g., the four-side model, Schulz von Thun, 1990) (week 2), preparing difficult conversations (week 3), and how to give constructive criticism (week 4). The trainings were performed under precautions such as wearing masks, limiting the numbers of participants per group, and physical distancing.

Four months later, two booster sessions were offered. The booster program included a 90 min (week 1) and a 30 min (week 2) face-to-face session with their previous trainer. The first session included an exchange of experience over the last 4 months and a new exercise (communication styles of Satir; Satir et al., 1991). The second session focused on active listening techniques.

### 2.4 Measures

#### 2.4.1 Mindfulness

Mindfulness was measured with the Five-Facet Mindfulness Questionnaire (Michalak et al., 2016) at T1 through T4. The questionnaire consists of 39 items and measures five facets of mindfulness (observing, describing, acting with awareness, non-judging, and non-reactivity; Baer et al., 2008). Every item is rated on a 5-point Likert scale (1 = *never true*; 5 = *always true*). Examples of the subscales are observing (e.g., “I pay attention to sensations, such as the wind in my hair or sun on my face,” α_T1_ = 0.75, α_T2_ = 0.73, α_T3_ = 0.78, and α_T4_ = 0.80), describing (“I can usually describe how I feel at the moment in considerable detail,” α_T1_ = 0.88, α_T2_ = 0.88, α_T3_ = 0.91, and α_T4_ = 0.90), acting with awareness (“I am easily distracted,” a reversed item, α_T1_ = 0.79, α_T2_ = 0.85, α_T3_ = 0.83, and α_T4_ = 0.85), non-judging (“I make judgments about whether my thoughts are good or bad,” a reversed item, α_T1_ = 0.91, α_T2_ = 0.94, α_T3_ = 0.94, and α_T4_ = 0.93), and non-reactivity (“When I have distressing thoughts or images, I just notice them and let them go,” α_T1_ = 0.84, α_T2_ = 0.83, α_T3_ = 0.85, and α_T4_ = 0.83).

#### 2.4.2 Mindful characteristics

Body awareness was assessed using the body awareness scale of the Scale of Body Connection (Price and Thompson, 2007) at T1 through T4. The body awareness scale consists of 12 items and measures the ability to experience inner body sensations (e.g., breathing, tension; “I notice that my breathing becomes shallow when I am nervous”) on a 5-point Likert scale (1 = *not at all*; 5 = *all of the time*). Cronbach’s α was α_T1_ = 0.74, α_T2_ = 0.83, α_T3_ = 0.87, and α_T4_ = 0.80.

Non-attachment was measured with the 7-item Non-attachment Scale (Sahdra et al., 2015) on a 6-point Likert scale (1 = *disagree strongly*; 6 = *agree strongly*) at T1 through T4. An example item is “I can let go of regrets and feelings of dissatisfaction about the past.” Internal consistency was at α_T1_ = 0.80, α_T2_ = 0.81, α_T3_ = 0.82, and α_T4_ = 0.75.

#### 2.4.3 Emotional regulation

Emotional regulation was assessed with three subscales (reappraisal, acceptance, rumination) from the Heidelberg Form for Emotion Regulation Strategies (Izadpanah et al., 2019). The subscales were used at T1 through T4. Each item was rated on a 5-point Likert scale (1 = *never*; 5 = *always*). Examples of the subscales are reappraisal (4 items, “When I want to feel better, I concentrate on the good aspects of a situation,” α_T1_ = 0.87, α_T2_ = 0.85, α_T3_ = 0.85, and α_T4_ = 0.74), acceptance (3 items, “When I cannot change something, I accept the situation as it is,” α_T1_ = 0.82, α_T2_ = 0.79, α_T3_ = 0.83, and α_T4_ = 0.83), and rumination (4 items, “I realise, again and again, that I have to think about something that made me angry or sad,” α_T1_ = 0.77, α_T2_ = 0.81, α_T3_ = 0.81, and α_T4_ = 0.78).

#### 2.4.4 Mental health

Depression was assessed with the German version of the Patient Health Questionnaire-8 scale (PHQ-8, Kroenke et al., 2009) at T1 through T4. The scale consists of eight items (e.g., “feeling down, depressed, or hopeless”). Each item is rated on a 4-point Likert scale (0 = *not at all*; 3 = *nearly every day*) that ranges from 0 to 24. Cronbach’s alpha was at α_T1_ = 0.85, α_T2_ = 0.83, α_T3_ = 0.85, and α_T4_ = 0.86.

Anxiety was assessed using the German version of the Generalized Anxiety Disorder scale (GAD-7, Spitzer et al., 2006) at T1 through T4. The GAD-7 consists of seven items scored from 0 (not at all) to 3 (nearly every day). The total score ranges between 0 and 21. An example item is “Feeling nervous, anxious, or on edge.” Internal consistency was high (α_T1_ = 0.88, α_T2_ = 0.85, α_T3_ = 0.88, α_T4_ = 0.83).

Stress was measured with the German version of the 10-item Perceived Stress Scale (PSS, Klein et al., 2016) on a 5-point Likert scale (0 = *never*; 4 = *very often*) at T1 through T4. The scale ranges from 0 to 40. An example item is “How often have you felt nervous and stressed?.” The reliability of the scale was high (α_T1_ = 0.86, α_T2_ = 0.82, α_T3_ = 0.88, α_T4_ = 0.88).

#### 2.4.5 Attentional abilities

The d2 attention test-Revision (d2-R) by Brickenkamp et al. (2010) was used to assess participants’ attentional abilities. The test was administered as a paper-and-pencil test at T1, T2, and T3. The d2 test is a time-limited test consisting of 14 lines with 57 characters per line (total: 798 items). The items consist of “d” and “p” with one, two, or no dashes above and/or below the letter. The participants must identify and cross out each letter “d” with two dashes. The time is limited to 20 s per line. The d2-R test shows a high test–retest reliability (> 0.90) with student samples (Brickenkamp et al., 2010). The following parameters were derived: the concentration performance, the working accuracy, and the working speed. The score concentration performance was obtained by the number of correctly crossed-out items minus the errors of commission. The working speed was computed as the sum of crossed-out items. The sum of all errors concerning working speed was used to estimate the working accuracy. The raw data were converted to age-specific standard values.

#### 2.4.6 Feasibility and acceptability

Feasibility and acceptability criteria were participants’ program satisfaction, course attendance, the frequency of meditation with the app 7Mind (both app groups), and home practice of formal mindfulness exercises (mindfulness and mindfulness+app group). The measures were addressed through self-report questionnaires and screenshots. Participants’ satisfaction with the mindfulness interventions or the communication course was assessed with four items (e.g., “I was satisfied with… (1) the trainer, (2) the climate of the course, (3) the design of the training course, and (4) the conditions of the course (time, room, etc.),” 1 = *not at all*; 6 = *absolutely;* α = 0.68; Karing and Beelmann, 2016). The number of face-to-face sessions attended was used to estimate participants’ course attendance. Furthermore, for the meditation frequency with the app 7Mind, the app group and the mindfulness+app group were asked to send a screenshot to the research team of the total number of minutes meditated report in the app 7Mind. The meditation frequency with the app 7Mind was assessed at T2, T3, and T4. However, only two participants sent the screenshot to the research team at T4. Thus, the results of screenshots are only reported for T2 and T3. Additionally, participants of the app groups were asked how often they used the app 7Mind after the intervention (1 = *never*, 5 = *always*). Furthermore, participants of the mindfulness and mindfulness+app group were also asked, every intervention week, how often they had practiced the formal mindfulness exercise at home in the past week. Participants’ continued practice of the taught mindfulness exercises was assessed at both follow-ups (1 = *not at all*, 4 = *nearly everything*). In addition, each trainer rated participants’ cooperation during the sessions (0 = *not at all*; 3 = *very much*).

### 2.5 Data analyses

First, multivariate analyses of variance (MANOVAs) using Pillai’s trace were performed to compare demographic characteristics (age, academic semester) and baseline measures at T1 between the four groups. Furthermore, a chi-square test was conducted to compare gender. In addition, MANOVAs were used to compare demographic characteristics (age, academic semester) and baseline measures at T1 between completers and non-completers who dropped out at T2.

To address the first research question, repeated measures analyses of variance (MANOVA, ANOVA), using Pillai’s trace, were performed to test for time effects, group effects, and the interaction between time and groups (intervention effects) across the four-time points (T1 and T2, T1 and T3, T1 and T4). All analyses were conducted using SPSS Statistics (Version 29, SPSS Inc., Chicago, IL, United States). Per-protocol (PP) analyses and intention-to-treat (ITT) analyses were conducted for all analyses (significance level was set at 0.05). Missing data in the ITT analyses were replaced by multiply-imputed datasets. ITT analyses were performed on all randomized students (*n =* 177, mindfulness group: *n* = 42, app group: *n* = 44, mindfulness+app group: *n* = 45, active control group: *n* = 46). PP analyses were carried out with 148 participants (T2), 110 participants (T3), and 86 participants (T4).

Regression analyses were performed to investigate the second research question on whether participants’ use of the app and/or formal mindfulness homework practice improved the change in outcome variables. Standardized residual change scores were used for each outcome variable by regressing the post-intervention scores onto the pre-intervention scores and the follow-up scores (T3) onto the pre-test scores. Next, regression analyses were calculated with residual change scores as the dependent variable. The predictor variables were app use and formal mindfulness home practice for the mindfulness+app group, app use for the app group, and formal mindfulness home practice for the mindfulness group.

## 3 Results

### 3.1 Demographic and baseline analysis

Table 2 (intention to treat) displays the descriptive statistics of all outcome measures in the pre-, post-intervention, and follow-ups for the four groups, and Supplementary Table S1 shows per-protocol descriptive statistics.

**Table 2:** Descriptive statistics by time and group (intention to treat).

	Pre-intervention (T1)				Post-intervention (T2)				Follow-up I (T3)				Follow-up II (T4)			
	Mindfulness +app	Mindfulness	App	Control	Mindfulness +app	Mindfulness	App	Control	Mindfulness +app	Mindfulness	App	Control	Mindfulness +app	Mindfulness	App	Control
Outcome	M (*SD*)	M (*SD*)	M (*SD*)	M (*SD*)	M (*SD*)	M (*SD*)	M (*SD*)	M (*SD*)	M (*SD*)	M (*SD*)	M (*SD*)	M (*SD*)	M (*SD*)	M (*SD*)	M (*SD*)	M (*SD*)
Mindfulness																
Observing	28.49 (6.05)	28.81 (4.53)	28.46 (5.82)	28.39 (5.57)	29.53 (4.25)	30.57 (3.79)	28.84 (4.34)	28.35 (4.45)	29.09 (4.90)	29.53 (3.98)	30.32 (3.73)	29.60 (3.80)	29.45 (4.27)	29.25 (3.77)	29.87 (3.09)	29.16 (3.67)
Describing	25.67 (7.33)	26.38 (6.10)	28.14 (6.40)	27.24 (6.52)	27.41 (6.58)	28.73 (5.78)	29.54 (4.95)	28.82 (5.07)	27.40 (7.25)	28.25 (5.72)	29.74 (4.55)	29.53 (4.27)	28.85 (5.89)	29.64 (4.65)	31.49 (3.67)	30.71 (3.24)
Acting awareness	24.76 (5.10)	22.38 (6.12)	23.71 (5.13)	24.02 (5.47)	24.72 (4.28)	24.49 (5.11)	24.68 (5.31)	23.89 (5.35)	26.42 (3.76)	24.48 (4.56)	25.25 (4.80)	25.43 (3.70)	24.85 (4.48)	24.88 (4.15)	24.21 (4.62)	24.76 (2.96)
Non-judging	24.09 (8.03)	24.93 (8.34)	25.16 (8.59)	24.74 (7.89)	26.72 (7.68)	27.09 (8.38)	28.83 (6.92)	26.15 (8.10)	29.39 (6.13)	29.25 (6.82)	28.26 (7.50)	27.13 (6.53)	26.05 (5.25)	27.91 (5.75)	26.54 (6.90)	27.30 (4.71)
Non-reactivity	18.87 (4.45)	17.79 (4.19)	17.05 (5.46)	18.72 (5.91)	20.55 (3.71)	20.61 (4.40)	19.82 (4.20)	20.57 (4.66)	20.67 (3.90)	20.47 (3.86)	21.02 (3.97)	20.95 (4.13)	19.99 (2.42)	21.32 (2.99)	20.39 (3.85)	20.97 (3.42)
Mindful characteristics																
Body awareness	3.45 (0.55)	3.58 (0.49)	3.49 (0.63)	3.57 (0.58)	3.71 (0.55)	3.85 (0.51)	3.73 (0.52)	3.73 (0.58)	3.60 (0.60)	3.75 (0.65)	3.82 (0.51)	3.65 (0.57)	3.86 (0.36)	3.86 (0.36)	3.89 (0.33)	3.74 (0.33)
Non-attachment	4.12 (0.84)	4.07 (0.94)	3.96 (1.01)	3.89 (0.89)	4.28 (0.76)	4.05 (0.94)	4.09 (0.82)	4.02 (0.80)	4.28 (0.72)	4.16 (0.94)	4.28 (0.77)	4.15 (0.70)	4.12 (0.46)	4.14 (0.55)	4.07 (0.57)	4.09 (0.51)
Emotional regulation																
Reappraisal	3.22 (0.83)	3.04 (0.88)	3.01 (0.92)	3.15 (0.94)	3.27 (0.79)	3.15 (0.87)	3.26 (0.63)	3.18 (0.87)	3.36 (0.69)	3.21 (0.85)	3.49 (0.62)	3.36 (0.69)	3.33 (0.54)	3.33 (0.57)	3.40 (0.46)	3.40 (0.54)
Acceptance	3.02 (0.91)	3.20 (1.01)	3.02 (0.99)	3.13 (1.00)	3.27 (0.73)	3.33 (0.90)	3.28 (0.84)	3.35 (0.80)	3.36 (0.69)	3.34 (0.75)	3.35 (0.78)	3.31 (0.82)	3.17 (0.69)	3.37 (0.74)	3.33 (0.60)	3.41 (0.63)
Rumination	4.02 (0.77)	4.07 (0.80)	4.00 (0.74)	3.90 (0.84)	3.74 (0.72)	3.82 (0.90)	3.61 (0.76)	3.59 (0.88)	3.55 (0.72)	3.59 (0.81)	3.68 (0.78)	3.56 (0.75)	3.74 (0.61)	3.72 (0.68)	3.75 (0.62)	3.67 (0.54)
Mental Health																
Depression	8.89 (4.48)	9.95 (6.24)	10.23 (5.17)	9.17 (4.91)	8.69 (4.38)	8.68 (5.08)	8.88 (4.43)	9.54 (4.30)	9.39 (4.03)	8.22 (3.76)	7.96 (3.13)	8.60 (2.75)	9.81 (3.78)	8.44 (3.73)	10.38 (3.91)	8.89 (2.89)
Anxiety	8.73 (5.22)	9.38 (5.64)	10.34 (4.55)	9.39 (4.66)	8.67 (4.15)	8.28 (5.05)	8.63 (3.98)	8.87 (3.87)	7.43 (3.47)	7.53 (3.51)	7.18 (2.26)	7.57 (2.67)	9.07 (3.19)	8.14 (3.37)	9.37 (3.42)	8.59 (2.28)
Stress	29.27 (6.86)	28.86 (7.42)	31.32 (6.80)	29.52 (6.75)	28.09 (6.15)	28.00 (6.42)	27.33 (6.69)	27.89 (6.10)	26.80 (6.25)	26.49 (6.69)	27.58 (5.95)	26.57 (4.91)	28.03 (4.96)	27.64 (4.40)	27.73 (4.82)	27.69 (4.81)
Attentional abilities																
Concentration	101.38 (11.28)	102.31 (8.75)	102.62 (8.05)	104.30 (9.10)	111.25 (8.72)	111.12 (9.07)	109.45 (7.19)	110.00 (8.40)	109.39 (2.11)	109.25 (5.85)	108.99 (2.98)	109.51 (4.06)	–	–	–	–
Working accuracy	101.69 (11.20)	102.00 (11.07)	103.90 (9.89)	102.39 (10.00)	109.03 (6.42)	108.63 (7.80)	109.08 (7.20)	108.09 (9.52)	106.54 (1.49)	106.01 (3.52)	106.59 (2.09)	105.60 (3.01)	–	–	–	–
Working speed	103.64 (11.49)	104.29 (11.48)	104.35 (11.55)	106.15 (10.35)	110.86 (9.15)	111.28 (11.22)	109.92 (9.56)	111.41 (7.93)	109.99 (2.35)	109.63 (6.17)	109.93 (3.46)	110.00 (4.21)	–	–	–	–

There were no differences between the four groups on age and academic semester [Pillai’s trace V = 0.03; *F*(6, 346) = 0.83, *p* = 0.549, η^2^ = 0.01] or gender [χ^2^(6) = 7.23, *p* = 0.300]. In addition, no differences between the groups were found on any baseline measure at T1 [Pillai’s trace V = 0.24; *F*(48, 471) = 0.85, *p* = 0.758, η^2^ = 0.079]. Furthermore, 47% of all participants reported moderate-to-severe symptoms of depression (PHQ-8 score ≥ 10), 42% showed moderate-to-severe symptoms of anxiety (GAD-7 ≥ 10), and 77% perceived high stress (PSS ≥ 25).

Analyses on completers and non-completers who dropped out at T2 did not show any differences in baseline measures at T1 [Pillai’s trace V = 0.08; *F*(16, 157) = 0.83, *p* = 0.648, η^2^ = 0.078], gender [χ^2^(2) = 0.71, *p* = 0.700], age and academic semester [Pillai’s Trace V = 0.02; *F*(2, 174) = 1.46, *p* = 0.236, η^2^ = 0.016].

### 3.2 Efficacy of the mindfulness interventions

The findings of the ITT repeated measures MANOVAs are presented in Table 3, and the results of the ITT repeated measures ANOVAs across the four-time points are displayed in Table 4. The results of the PP repeated measures MANOVAs and PP repeated measures ANOVAs can be found in Supplementary Tables S2, S3.

**Table 3:** Results of the ITT repeated measures MANOVAs.

	Pre-intervention – post-intervention (T1 – T2)						Pre-intervention – follow-up I (T1 – T3)						Pre-intervention – follow-up II (T1 – T4)					
Outcome	*V*	*F*	*df1*	*df2*	*p*	*η_p_^2^*	*V*	*F*	*df1*	*df2*	*p*	*η_p_^2^*	*V*	*F*	*df1*	*df2*	*p*	*η_p_^2^*
**Mindfulness**																		
Group effect	0.10	1.13	15	513	0.323	0.03	0.12	1.44	15	513	0.126	0.04	0.11	1.34	15	513	0.174	0.04
Time effect	0.30	14.33	5	169	**<0.001**	0.30	0.33	16.54	5	169	**<0.001**	0.33	0.30	14.60	5	169	**<0.001**	0.30
Interaction effect	0.09	1.01	15	513	0.440	0.03	0.08	0.96	15	513	0.493	0.03	0.08	0.88	15	513	0.582	0.03
**Mindfulness characteristics**																		
Group effect	0.03	0.76	6	346	0.601	0.01	0.02	0.69	6	346	0.654	0.01	0.02	0.45	6	346	0.843	0.01
Time effect	0.18	18.95	2	172	**<0.001**	0.18	0.12	11.70	2	172	**<0.001**	0.12	0.23	25.68	2	172	**<0.001**	0.23
Interaction effect	0.02	0.51	6	346	0.802	0.01	0.03	0.97	6	346	0.448	0.02	0.04	1.08	6	346	0.375	0.02
**Emotional regulation**																		
Group effect	0.04	0.68	9	519	0.724	0.01	0.03	0.51	9	519	0.868	0.01	0.03	0.61	9	519	0.789	0.01
Time effect	0.19	13.07	3	171	**<0.001**	0.19	0.25	19.27	3	171	**<0.001**	0.25	0.16	10.93	3	171	**<0.001**	0.16
Interaction effect	0.02	0.38	9	519	0.944	0.01	0.05	0.91	9	519	0.519	0.02	0.02	0.49	9	519	0.914	0.01
**Mental health**																		
Group effect	0.01	0.24	9	519	0.988	0.00	0.04	0.82	9	519	0.603	0.01	0.03	0.50	9	519	0.877	0.01
Time effect	0.09	5.88	3	171	**<0.001**	0.09	0.21	15.03	3	171	**<0.001**	0.21	0.08	5.01	3	171	**0.002**	0.08
Interaction effect	0.08	1.49	9	519	0.148	0.03	0.06	1.12	9	519	0.348	0.02	0.07	1.39	9	519	0.190	0.02
**Attentional abilities**																		
Group effect	0.02	0.36	9	519	0.956	0.01	0.04	0.85	9	519	0.569	0.02	–	–	–	–	–	–
Time effect	0.57	76.08	3	171	**<0.001**	0.57	0.36	31.48	3	171	**<0.001**	0.36	–	–	–	–	–	–
Interaction effect	0.06	1.27	9	519	0.253	0.02	0.02	0.33	9	519	0.966	0.01	–	–	–	–	–	–

Bold values represent the statistically significant results.

**Table 4:** Results of the ITT repeated measures ANOVAs.

	Pre-intervention – post-intervention (T1 – T2)									Pre-intervention – follow-up I (T1 – T3)								
	Group effect			Time effect			Interaction effect			Group effect			Time effect			Interaction effect		
Outcome	*F*	*p*	*η_p_^2^*	*F*	*p*	*η_p_^2^*	*F*	*p*	*η_p_^2^*	*F*	*p*	*η_p_^2^*	*F*	*p*	*η_p_^2^*	*F*	*p*	*η_p_^2^*
**Mindfulness**																		
Observing	0.73	0.535	0.01	5.47	**0.020**	0.03	1.35	0.261	0.02	0.17	0.915	0.00	7.56	**0.007**	0.04	0.52	0.670	0.01
Describing	1.31	0.273	0.02	21.94	**<0.001**	0.11	0.29	0.836	0.01	1.79	0.151	0.03	18.13	**<0.001**	0.10	0.12	0.951	0.00
Acting awareness	0.58	0.628	0.01	4.17	**0.043**	0.02	2.12	0.099	0.04	1.99	0.117	0.03	18.95	**<0.001**	0.10	0.15	0.932	0.00
Non-judging	0.45	0.717	0.01	28.20	**<0.001**	0.14	1.05	0.373	0.02	0.24	0.869	0.00	51.24	**<0.001**	0.23	1.53	0.210	0.03
Non-reactivity	0.85	0.466	0.02	58.58	**<0.001**	0.25	1.01	0.390	0.02	0.53	0.666	0.01	54.33	**<0.001**	0.24	1.68	0.174	0.03
**Mindfulness characteristics**																		
Body awareness	0.59	0.621	0.01	37.35	**<0.001**	0.18	0.44	0.726	0.01	0.77	0.512	0.01	16.93	**<0.001**	0.09	1.40	0.244	0.02
Non-attachment	0.77	0.511	0.01	3.34	0.070	0.02	0.49	0.689	0.01	0.48	0.694	0.01	10.44	**0.001**	0.06	0.62	0.605	0.01
**Emotional regulation**																		
Reappraisal	0.32	0.812	0.01	3.27	0.072	0.02	0.67	0.575	0.01	0.45	0.715	0.01	16.07	**<0.001**	0.09	1.58	0.196	0.03
Acceptance	0.29	0.833	0.01	8.95	**0.003**	0.05	0.15	0.928	0.00	0.12	0.947	0.00	11.83	**<0.001**	0.06	0.51	0.677	0.01
Rumination	0.63	0.594	0.01	37.59	**<0.001**	0.18	0.38	0.769	0.01	0.27	0.845	0.01	45.68	**<0.001**	0.21	0.52	0.669	0.01
**Mental health**																		
Depression	0.26	0.857	0.00	3.20	0.075	0.02	1.50	0.215	0.03	0.04	0.988	0.00	7.01	**0.009**	0.04	2.59	0.054	0.04
Anxiety	0.30	0.824	0.01	7.74	**0.006**	0.04	1.38	0.250	0.02	0.31	0.815	0.01	30.19	**<0.001**	0.15	1.14	0.335	0.02
Stress	0.18	0.911	0.00	17.35	**<0.001**	0.09	2.37	0.072	0.04	0.86	0.463	0.02	33.95	**<0.001**	0.16	0.40	0.755	0.01
**Attentional abilities**																		
Concentration	0.16	0.923	0.00	211.35	**<0.001**	0.55	3.16	0.026	0.05	0.63	0.599	0.01	93.86	**<0.001**	0.35	0.75	0.525	0.01
Working accuracy	0.24	0.866	0.00	78.10	**<0.001**	0.31	0.47	0.703	0.01	0.49	0.690	0.01	23.01	**<0.001**	0.12	0.38	0.766	0.01
Working speed	0.29	0.835	0.01	92.39	**<0.001**	0.35	0.58	0.627	0.01	0.33	0.802	0.01	44.27	**<0.001**	0.20	0.45	0.719	0.01

	Pre-intervention – follow-up II (T1 – T4)								
	Group effect			Time effect			Interaction effect		
Outcome	*F*	*p*	*η_p_^2^*	*F*	*p*	*η_p_^2^*	*F*	*p*	*η_p_^2^*
**Mindfulness**									
Observing	0.08	0.969	00	4.29	**0.040**	0.02	0.22	0.883	0.00
Describing	2.51	0.061	0.04	49.27	**<0.001**	0.22	0.02	0.997	0.00
Acting awareness	0.78	0.504	0.01	4.62	**0.033**	0.03	1.37	0.253	0.02
Non-judging	0.42	0.737	0.01	13.23	**<0.001**	0.07	0.32	0.810	0.01
Non-reactivity	0.86	0.465	0.02	47.39	**<0.001**	0.22	2.24	0.085	0.04
**Mindfulness characteristics**									
Body awareness	0.34	0.796	0.01	51.40	**<0.001**	0.23	1.65	0.180	0.03
Non-attachment	0.58	0.630	0.01	1.82	0.180	0.01	0.37	0.774	0.01
**Emotional regulation**									
Reappraisal	0.28	0.843	0.01	15.93	**<0.001**	0.08	0.75	0.522	0.01
Acceptance	0.76	0.517	0.01	9.26	**0.003**	0.05	0.29	0.837	0.01
Rumination	0.36	0.785	0.01	19.25	**<0.001**	0.10	0.19	0.900	0.00
**Mental health**									
Depression	1.09	0.356	0.02	0.21	0.648	0.00	1.65	0.181	0.03
Anxiety	0.94	0.422	0.02	3.30	0.071	0.02	0.89	0.447	0.02
Stress	0.58	0.626	0.01	12.67	**<0.001**	0.07	1.01	0.391	0.02
**Attentional abilities**									
Concentration	–	–	–	–	–	–	–	–	–
Working accuracy	–	–	–	–	–	–	–	–	–
Working speed	–	–	–	–	–	–	–	–	–

Bold values represent the statistically significant results.

#### 3.2.1 Mindfulness

Concerning the five facets of mindfulness, PP and ITT repeated measures MANOVAs from T1 to T2 showed a significant time effect. However, the group effect and the interaction time x group were not significant. Subsequent repeated measures of ANOVAs showed a significant time effect for non-judging, describing, and non-reactivity in the PP sample and all mindfulness facets in the ITT sample. Based on the intention-to-treat findings, there was an increase in observing, describing, non-judging, and non-reactivity in each intervention group. Acting with awareness increased only in the mindfulness group and app group. Furthermore, describing, non-judging, and non-reactivity also increased over time in the active control group.

Both repeated measures of MANOVAs from T1 to T3 and T1 to T4 yielded significant time effects. Furthermore, the group effect at T3 and T4 was significant in the PP analyses but was not significant in the ITT analyses. For both follow-ups, there was no significant interaction between time and group for any mindfulness facets. Subsequent repeated measures of ANOVAs showed a significant time effect for describing, non-judging, and non-reactivity in the PP sample and a significant time effect for all mindfulness facets in the ITT sample. Based on the ITT results, all mindfulness facets improved for all groups (intervention and control groups) from T1 to T3 and from T1 to T4. There was no significant group effect on any variable at T3 (PP and ITT samples). A significant group effect was only observed for describing (*p* < 0.05) at T4 in the PP sample but not for the other mindfulness facets.

#### 3.2.2 Mindful characteristics

The repeated measures MANOVA using body awareness and non-attachment from T1 to T2 showed a significant main effect of time across the PP and ITT samples. The main impact of the group and interaction effects was statistically insignificant in the PP and ITT samples. Repeated measures ANOVAs showed that only body awareness, the main effect of time, was significant in both analyses (PP and ITT). The findings indicated that all groups (intervention and control groups) improved in body awareness from T1 to T2.

The repeated measures MANOVAs revealed significant time, but not group, effects at both follow-ups in the PP and ITT samples. Body awareness was higher for all groups at T3 and T4 than at T1. Non-attachment was higher for all groups only at T3 than at T1. Furthermore, a significant interaction between time and group was only found for body awareness at T3 in the PP sample but not in the ITT sample.

#### 3.2.3 Emotional regulation

The results of repeated measures MANOVAs in PP and ITT samples showed significant main effects of time from T1 to T2. The main effect of the group and the interaction between time and group became insignificant. Univariate analyses showed a significant time effect for acceptance and rumination across the PP and ITT samples. The findings indicated that students in all intervention groups and the active control group improved on acceptance and rumination from T1 to T2. However, there was no significant effect of time for reappraisal, when comparing T1 to T2 for both PP and ITT analyses.

The repeated measures MANOVAs from T1 to T3 and T1 to T4 yielded significant main effects of time (PP and ITT samples). However, neither was a significant group effect or time x group interaction from pre-intervention to follow-ups in both samples (PP and ITT). Univariate analyses showed a significant time effect for acceptance, reappraisal, and rumination at T3 and T4, except for acceptance and reappraisal at T4 in the PP sample. Based on the ITT results, all emotion regulation strategies improved for all groups (intervention and control groups) from T1 to T3 and from T1 to T4.

#### 3.2.4 Mental health

PP and ITT repeated measures MANOVAs from T1 to T2 showed a significant main effect of time. However, the group effect and the interaction time x group were not significant. Using repeated measures ANOVA, a significant time effect for stress, anxiety, and depression was found, except for depression in the ITT sample. Based on the ITT results, stress and anxiety declined for all intervention groups and the active control group from T1 to T2.

The results of repeated measures MANOVAs showed significant time effects from T1 to T3 in the PP and ITT samples, and from T1 to T4 (only in the ITT sample). The group effect and the interaction between time and group were insignificant. Univariate analyses revealed a significant time effect for stress, anxiety, and depression at T3 (PP and ITT samples). Based on the ITT results, stress and anxiety were lower for all groups at T3 than at T1. However, only the time effect for stress stayed significant at T4 in the ITT sample but not in the PP sample.

#### 3.2.5 Attentional abilities

Repeated measures MANOVA results indicated a significant time effect from T1 to T2 in the PP and ITT samples. However, the group effect and the interaction time x group were not significant. A significant time effect for concentration performance, working accuracy, and working speed was found in the PP and ITT samples using repeated measures analyses of variance. Concentration performance, working accuracy, and working speed improved for all groups (intervention and control groups) from T1 to T2.

The repeated measures MANOVAs revealed no group effects and time x group interactions from T1 to T3 in the PP and ITT samples. However, there were significant time effects. Univariate analyses showed a significant time effect for concentration performance, working accuracy, and working speed in the ITT sample; however, the time effect for working accuracy was insignificant in the PP sample. Based on the results of the ITT analyses, the concentration performance, working accuracy, and working speed improved for all intervention groups and the active control group from T1 to T3.

### 3.3 Feasibility and acceptability

Both mindfulness groups were very satisfied with the training (mindfulness group: *M* = 5.55, *SD* = 0.42; mindfulness+app group: *M* = 5.56, *SD* = 0.49). Furthermore, participants of the active control group were also very satisfied with their course (*M* = 5.27, *SD* = 0.62). There was no significant difference between the three groups regarding course satisfaction [*F*(2, 74) = 2.75, *p* = 0.071].

Regarding course attendance, students of the mindfulness group attended 3.09 of four sessions (*SD* = 0.93), and participants of the mindfulness+app group attended 3.11 of four sessions (*SD* = 0.96). The average number of sessions attended for the active control group was 3.00 (*SD* = 1.19). There was no significant difference between the groups for course attendance [*F*(2, 120) = 0.15, *p* = 0.865].

Furthermore, the frequency of app use was assessed with screenshots of the total number of minutes meditated report in the app 7Mind. On average, the app group used the app 7Mind throughout the 4 weeks of the intervention, 71.45 min (*SD* = 25.93), whereas the mindfulness+app group used the app 55.91 min (*SD* = 23.24). The difference was significant (*t* = 2.98, *p* = 0.004). Throughout the 4 months after the intervention (T3), the mean meditation frequency with the app 7Mind was only 38.88 min (*SD* = 41.72) for the app group and 48.69 min (*SD* = 102.96) for the mindfulness+app group. The difference was insignificant (*t* = 0.59, *p* = 0.279). One year after the intervention, of the 37 students of the app groups who completed the second follow-up survey, 48.6% reported that they did not use the app 7Mind anymore (app group, *n* = 19: 42.1%, mindfulness+app group, *n* = 18: 55.5%), and 27% reported that they used the app 7Mind rarely (app group: 36.8%, mindfulness+app group: 16.7%). The main reasons for not using the app 7Mind after the intervention were “not interested anymore” (31.3%), lack of time (18.8%), and other issues (31.3%, e.g., only a few exercises were free of charge).

Regarding the home practice of formal mindfulness exercises during the entire intervention, the mean rate for the mindfulness group was 3.34 (*SD* = 2.11) and 3.62 (*SD* = 2.39) for the mindfulness+app group. The results show no significant difference between the groups (*t* = 0.58, *p* = 0.315). Four months after the intervention, 86.6% of the mindfulness+app group and 96.6% of the mindfulness group reported still practicing formal mindfulness exercises. Similar findings were observed 1 year after the intervention. In total, 83.3% of the mindfulness+app group and 91.3% of the mindfulness group reported still practicing formal mindfulness exercises.

In addition, the trainers stated that students in each mindfulness group and the active control group were, on average, very cooperative during the sessions (mindfulness group: *M* = 2.64, *SD* = 0.43; mindfulness+app group: *M* = 2.61, *SD* = 0.62; active control group: *M* = 2.69, *SD* = 0.45).

### 3.4 App use, homework compliance, and change in intervention outcomes

Regression analyses (see Tables 5, 6) showed for the mindfulness+app group that a greater frequency of formal mindfulness home practice during the intervention predicted a more considerable decrease in depression and stress from T1 to T2, but this did not last through to T3 (4 months after intervention). However, a greater app use of 7Mind during the intervention predicted a greater increase in stress from T1 to T2 among the mindfulness+app group. On the other hand, a greater app use of 7Mind during the intervention predicted greater improvements in body awareness. Furthermore, a greater app use during the 4 months after the intervention predicted improvements in rumination, acceptance, and acting with awareness from T1 to T3.

**Table 5:** Results of the regression analyses: pre-test – post-test (T1 – T2).

	Mindfulness + app group					Mindfulness group					App group				
Outcome	*B*	*SE*	*ß*	*t*	*p*	*B*	*SE*	*ß*	*t*	*p*	*B*	*SE*	*ß*	*t*	*p*
**Mindfulness**															
Observing															
App 7Mind use	0.01	0.01	0.13	0.85	0.202	–	–	–	–	–	0.00	0.01	0.09	0.59	0.279
Form. homework	0.02	0.07	0.05	0.32	0.377	0.09	0.06	0.22	1.45	0.078	–	–	–	–	–
Describing															
App 7Mind use	0.00	0.01	−0.01	−0.06	0.477	–	–	–	–	–	0.01	0.01	0.23	1.53	0.068
Form. homework	0.02	0.07	0.04	0.22	0.413	0.17	0.05	0.44	3.05	**0.002**	–	–	–	–	–
Acting awareness															
App 7Mind use	0.00	0.01	−0.06	−0.40	0.346	–	–	–	–	–	0.01	0.01	0.30	2.05	**0.023**
Form. homework	0.02	0.06	0.06	0.40	0.347	0.03	0.06	0.08	0.53	0.300	–	–	–	–	–
Non-judging															
App 7Mind use	0.01	0.01	0.24	1.59	0.060	–	–	–	–	–	0.00	0.01	0.01	0.03	0.488
Form. homework	0.03	0.06	0.07	0.49	0.316	0.05	0.06	0.12	0.78	0.220	–	–	–	–	–
Non-reactivity															
App 7Mind use	0.00	0.01	0.02	0.11	0.456	–	–	–	–	–	0.00	0.01	−0.04	−0.27	0.395
Form. homework	0.02	0.07	0.05	0.32	0.377	−0.05	0.07	−0.10	−0.64	0.262	–	–	–	–	–
**Mindfulness characteristics**															
Body awareness															
App 7Mind use	0.01	0.01	0.28	1.91	**0.032**	–	–	–	–	–	0.01	0.01	0.27	1.83	**0.037**
Form. homework	0.09	0.06	0.23	1.55	0.065	0.10	0.07	0.23	1.51	0.070	–	–	–	–	–
Non-attachment															
App 7Mind use	0.00	0.01	−0.04	−0.25	0.402	–	–	–	–	–	0.00	0.01	0.02	0.11	0.458
Form. homework	0.05	0.06	0.12	0.77	0.223	0.05	0.07	0.12	0.79	0.216	–	–	–	–	–
**Emotional regulation**															
Reappraisal															
App 7Mind use	0.00	0.01	−0.06	−0.40	0.346	–	–	–	–	–	−0.01	0.01	−0.15	−0.96	0.171
Form. homework	−0.05	0.07	−0.11	−0.68	0.249	−0.03	0.08	−0.06	−0.35	0.365	–	–	–	–	–
Acceptance															
App 7Mind use	0.00	0.01	0.02	0.14	0.445	–	–	–	–	–	0.00	0.01	0.08	0.50	0.309
Form. homework	0.05	0.07	0.13	0.80	0.213	0.04	0.07	0.09	0.58	0.282	–	–	–	–	–
Rumination															
App 7Mind use	0.01	0.01	0.22	1.40	0.084	–	–	–	–	–	0.00	0.01	−0.01	−0.07	0.474
Form. homework	−0.06	0.07	−0.13	−0.85	0.202	0.07	0.06	0.18	1.16	0.127	–	–	–	–	–
**Mental health**															
Depression															
App 7Mind use	0.01	0.01	0.17	1.13	0.133	–	–	–	–	–	−0.01	0.01	−0.25	−1.68	0.051
Form. homework	−0.17	0.07	−0.35	−2.35	**0.012**	0.01	0.07	0.01	0.07	0.471	–	–	–	–	–
Anxiety															
App 7Mind use	0.01	0.01	0.11	0.73	0.235	–	–	–	–	–	0.00	0.01	0.03	0.21	0.416
Form. homework	−0.09	0.07	−0.19	−1.24	0.111	0.12	0.07	0.26	1.67	0.052	–	–	–	–	–
Stress															
App 7Mind use	0.01	0.01	0.26	1.71	**0.047**	–	–	–	–	–	0.01	0.01	0.27	1.84	**0.037**
Form. homework	−0.13	0.06	−0.30	−1.99	**0.027**	−0.06	0.06	−0.17	−1.07	0.145	–	–	–	–	–
**Attentional abilities**															
Concentration															
App 7Mind use	−0.01	0.01	−0.20	−1.29	0.102	–	–	–	–	–	0.00	0.01	−0.05	−0.29	0.386
Form. homework	−0.09	0.08	−0.16	−1.04	0.152	−0.03	0.07	−0.07	−0.43	0.337	–	–	–	–	–
Working accuracy															
App 7Mind use	−0.01	0.01	−0.18	−1.18	0.123	–	–	–	–	–	0.01	0.01	0.14	0.89	0.189
Form. homework	0.01	0.06	0.02	0.10	0.460	−0.04	0.07	−0.10	−0.61	0.274	–	–	–	–	–
Working Speed															
App 7Mind use	−0.03	0.01	−0.08	−0.51	0.308	–	–	–	–	–	0.00	0.01	−0.01	−0.09	0.463
Form. homework	−0.04	0.07	−0.10	−0.65	0.260	−0.04	0.07	−0.09	−0.60	0.277	–	–	–	–	–

Form. Homework, formal mindfulness home practice during the intervention; app use during the intervention. Bold values represent the statistically significant results.

**Table 6:** Results of the regression analyses: pre-test – follow-up I (T1 – T3).

	Mindfulness + app group					Mindfulness group					App group				
Outcome	*B*	*SE*	*ß*	*t*	*p*	*B*	*SE*	*ß*	*t*	*p*	*B*	*SE*	*ß*	*t*	*p*
**Mindfulness**															
Observing															
App 7Mind use	0.00	0.00	0.01	0.03	0.487	–	–	–	–	–	0.00	0.00	−0.12	−0.77	0.224
Form. homework	−0.11	0.08	−0.20	−1.32	0.097	−0.02	0.06	−0.05	−0.33	0.373	–	–	–	–	–
Describing															
App 7Mind use	0.00	0.00	0.20	1.32	0.098	–	–	–	–	–	0.00	0.00	0.06	0.37	0.356
Form. homework	0.04	0.08	0.07	0.49	0.315	0.04	0.07	0.10	0.62	0.271	–	–	–	–	–
Acting awareness															
App 7Mind use	0.00	0.00	0.28	1.92	**0.031**	–	–	–	–	–	0.00	0.00	0.13	0.82	0.208
Form. homework	−0.01	0.06	−0.03	−0.20	0.423	−0.03	0.07	−0.07	−0.45	0.329	–	–	–	–	–
Non-judging															
App 7Mind use	0.00	0.00	−0.01	−0.08	0.470	–	–	–	–	–	0.00	0.00	−0.06	−0.40	0.345
Form. homework	0.03	0.07	0.07	0.47	0.320	0.04	0.06	0.11	0.70	0.245	–	–	–	–	–
Non-reactivity															
App 7Mind use	0.00	0.00	0.18	1.16	0.127	–	–	–	–	–	0.00	0.00	0.17	1.09	0.141
Form. homework	0.04	0.08	0.07	0.49	0.314	−0.12	0.06	−0.29	−1.94	**0.030**	–	–	–	–	–
**Mindfulness characteristics**															
Body awareness															
App 7Mind use	0.00	0.00	0.12	0.81	0.212	–	–	–	–	–	0.00	0.00	−0.09	−0.57	0.287
Form. homework	−0.01	0.07	−0.02	−0.13	0.450	0.06	0.07	0.13	0.84	0.205	–	–	–	–	–
Non-attachment															
App 7Mind use	0.00	0.00	0.25	1.65	0.053	–	–	–	–	–	0.00	0.00	0.04	0.23	0.408
Form. homework	−0.03	0.07	−0.07	−0.48	0.318	−0.09	0.07	−0.21	−1.33	0.097	–	–	–	–	–
**Emotional regulation**															
Reappraisal															
App 7Mind use	0.00	0.00	0.17	1.15	0.129	–	–	–	–	–	0.00	0.00	0.07	0.46	0.325
Form. homework	−0.11	0.07	−0.24	−1.62	0.057	−0.07	0.07	−0.15	−0.98	0.166	–	–	–	–	–
Acceptance															
App 7Mind use	0.00	0.00	0.28	1.89	**0.033**	–	–	–	–	–	0.00	0.00	0.11	0.73	0.234
Form. homework	0.06	0.07	0.12	0.83	0.207	−0.11	0.06	−0.27	−1.78	**0.042**	–	–	–	–	–
Rumination															
App 7Mind use	0.00	0.00	−0.29	−1.94	**0.030**	–	–	–	–	–	0.00	0.00	−0.06	−0.39	0.349
Form. homework	−0.06	0.06	−0.14	−0.91	0.184	0.08	0.07	0.20	1.30	0.100	–	–	–	–	–
**Mental health**															
Depression															
App 7Mind use	0.00	0.00	−0.23	−1.55	0.065	–	–	–	–	–	0.01	0.00	0.31	2.14	**0.019**
Form. homework	−0.02	0.08	−0.03	−0.20	0.420	−0.11	0.07	−0.25	−1.60	0.059	–	–	–	–	–
Anxiety															
App 7Mind use	0.00	0.00	−0.17	−1.09	0.141	–	–	–	–	–	0.00	0.00	0.20	1.34	0.094
Form. homework	0.02	0.08	0.03	0.22	0.413	−0.05	0.08	−0.11	−0.67	0.253	–	–	–	–	–
Stress															
App 7Mind use	0.00	0.00	−0.23	−1.49	0.072	–	–	–	–	–	0.00	0.00	0.00	0.01	0.498
Form. homework	−0.02	0.07	−0.04	−0.29	0.386	−0.13	0.07	−0.29	−1.90	**0.033**	–	–	–	–	–
**Attentional abilities**															
Concentration															
App 7Mind use	0.00	0.00	0.14	0.89	0.189	–	–	–	–	–	0.00	0.00	0.14	0.89	0.190
Form. homework	0.00	0.04	0.00	−0.01	0.496	0.07	0.09	0.12	0.74	0.232	–	–	–	–	–
Working accuracy															
App 7Mind use	0.00	0.00	−0.04	−0.24	0.408	–	–	–	–	–	0.00	0.00	−0.20	−1.32	0.097
Form. homework	−0.07	0.04	−0.24	−1.62	0.057	−0.02	0.09	−0.03	−0.20	0.421	–	–	–	–	–
Working Speed															
App 7Mind use	0.00	0.00	−0.09	−0.57	0.285	–	–	–	–	–	0.01	0.00	0.22	1.45	0.078
Form. homework	0.00	0.05	0.00	−0.01	0.496	0.06	0.09	0.11	0.68	0.251	–	–	–	–	–

Form. Homework, formal mindfulness home practice after the intervention; app use after the intervention. Bold values represent the statistically significant results.

A similar pattern was found for stress and body awareness from pre- to post-test in the app group. A greater app use of 7Mind during the intervention predicted a greater worsening of stress symptoms. By contrast, a greater app use during the intervention predicted a greater increase in body awareness. Furthermore, a greater app use during the 4 months after the intervention predicted a larger increase in depression from T1 to T3.

In the mindfulness group, a greater frequency of formal mindfulness home practice during the intervention predicted only an increase in the mindfulness facet described from pre- to post-test. However, this did not last through to T3. In addition, a greater frequency of mindfulness home practice predicted more significant stress improvements from T1 to T3.

## 4 Discussion

The study investigated the short-medium-long-term efficacy of a combined mindfulness intervention (face-to-face intervention plus app), a face-to-face mindfulness intervention alone, and a mindfulness app alone, compared with an active control group during the COVID-19 pandemic in Germany. Repeated nationwide lockdowns and contact restrictions during this time massively affected students’ academic and private lives, financial situations, and mental health (e.g., Karing, 2021; Appleby et al., 2022). We found that 47% of the students showed moderate-to-severe symptoms of depression, 42% reported moderate-to-severe symptoms of anxiety, and 77% perceived high stress at T1. These rates of depression, anxiety, and stress were notably higher than those found in our previous studies with students during the early phase of the COVID-19 pandemic (Karing, 2021, 2023).

The study’s findings showed that participants in the combined mindfulness intervention did not outperform students in the single interventions or the active control group in mindfulness, mindful characteristics, emotion regulation, mental health, and attentional abilities in the short, medium, and long term. Furthermore, there was no significant difference between the three intervention groups and the active control group on all measures. Thus, the results did not support the hypotheses. Although there was no difference between the groups, the findings showed that all intervention groups improved in mindfulness facets (observing, describing, non-judging, and non-reactivity), body awareness, emotion regulation strategies (acceptance, rumination), stress, and attentional abilities in short, medium, and long term. However, the active control group also showed improvements on these measures in the short term (except on mindfulness facet observing and acting with awareness) and in the medium and long term. The findings fit into the results from earlier studies with student samples (e.g., Josefsson et al., 2014; Sun et al., 2022). Josefsson et al. (2014) found no differences in all mindfulness facets, coping skills, and attention between the mindfulness and relaxation groups in their mindfulness intervention study. The mindfulness facets, coping skills, and attention improved in both groups over the intervention period. Furthermore, Johnson et al. (2023) reported in their meta-analysis that mindfulness training did not improve mindfulness, emotion regulation, or attention control compared to active control groups. Similar findings were reported for online mindfulness interventions during the COVID-19 pandemic. Sun et al. (2022) and Lahtinen et al. (2023) showed that the mindfulness group did not outperform the active control group on mindfulness. There was an improvement in mindfulness in both groups. A possible reason for the absence of a unique mindfulness intervention effect on mindfulness could be that non-mindfulness interventions (active control group) include similar exercises as mindfulness interventions (Josefsson et al., 2014). The active control group of our study was a communication training, which included exercises such as communication basics, preparing difficult conversations, and giving constructive criticism. These exercises also involved verbalizing feelings, thoughts and opinions, being calm, non-reactive, and non-judging. Thus, the communication training might also teach mindfulness skills. Another reason could be that some trainers of non-mindfulness interventions teach mindfully (Xia et al., 2019). Furthermore, Goldberg et al. (2019) argue that the responsiveness of self-report measures of mindfulness to non-mindfulness-based interventions might reflect construct-irrelevant variance.

Regarding mental health issues, both the intervention groups and the active control group improved in anxiety in the short and medium term. For depression, there was only a significant time effect 4 months after the intervention. The findings are consistent with the results from several reviews and meta-analyses showing that online and face-to-face mindfulness interventions with university students did not outperform active control groups for mental health issues (e.g., Halladay et al., 2019; Dawson et al., 2020; Alrashdi et al., 2023; Johnson et al., 2023). However, most of these studies conducted only short-term follow-ups. Furthermore, Josefsson et al. (2014) found that both mindfulness intervention and active control group (relaxation training) improved anxiety and depression in the short term. Thus, active interventions such as communication and relaxation training may be helpful as mindfulness interventions for improving students’ mental health issues (e.g., Josefsson et al., 2014; Dawson et al., 2020). Our findings propose that the social interaction with the trainer and the other participants during the communication course could contribute to the beneficial effects, especially on anxiety. Thus, the group aspect of the intervention may have positively impacted anxiety, especially at a time when there were many restrictions on meeting in groups. However, the long-term results of this study showed for all groups (mindfulness and communication interventions) that there was no significant reduction in depression and anxiety 1 year after the interventions. The length of these 4-week mindfulness and communication interventions seems to be too short to elicit substantial improvements in students’ depression and anxiety in the long term. This indicates that other strategies, such as ongoing booster sessions, are needed to maintain improvements in depression and anxiety in the long term. Although booster sessions were offered to all participants in this study after 4 months, only 16.9% of participants attended the booster sessions. Similar participation rates in booster sessions were reported by other intervention studies (e.g., van Son et al., 2014). Thus, strategies for increasing participation in booster sessions (e.g., using incentives) and research on the efficacy of booster sessions on long-term intervention outcomes are needed.

Although a greater app use of 7Mind during the intervention predicted a greater increase in body awareness in both app groups, a greater app use was also associated with a greater worsening of stress symptoms, thus indicating that increasing app use can negatively affect students’ health outcomes. More research is necessary on the critical amount of time spent using a mindfulness app for health outcomes. Furthermore, formal mindfulness practice at home during the intervention was associated with positive changes in depression and stress. A similar relation was also found for stress after the end of the intervention in the mindfulness group. Similar results were reported in a meta-analysis by Parsons et al. (2017), where formal home practice was positively related to improvements in intervention outcomes (e.g., mental health). Thus, formal home practice can positively affect changes in mental health outcomes. Birtwell et al. (2019) identified several supportive factors (e.g., practical resources, time and routine, support from others, and attitudes) for maintaining formal mindfulness practice. Future mindfulness intervention studies should also pay attention to such supportive factors.

## 5 Limitations

Several limitations of the present study should be considered. The majority of the students were female participant. However, several meta-analyses have reported that over-representing female participants is typical in mindfulness intervention studies (e.g., Jayawardene et al., 2017). Nevertheless, a meta-analysis by de Vibe et al. (2017) showed that the proportion of female participants in the sample did not correlate with the effect size magnitude for mental health. In addition, the sample size may be limited because of the dropouts at T2, T3, and T4, which reduced the initially planned sample size and could reduce the power of the study. Most of the measures were self-reported questionnaires and thus were subject to response bias. Future studies should include more comprehensive assessments of mental health issues (e.g., diagnostic assessments, Sun et al., 2022). Furthermore, the pre-test scores of depression, anxiety, and stress were higher than those found in our previous studies with students during the early phase of the COVID-19 pandemic (Karing, 2021, 2023). Almost half of the participants showed clinically relevant symptoms of depression and anxiety. In addition, 77% perceived high stress. These findings may suggest that, in particular, students with symptoms of depression, anxiety, and high stress were more interested in taking part in low-threshold interventions that were available during the COVID-19 pandemic. However, such low-threshold interventions should only complement and not replace psychotherapeutic treatments in students with clinically relevant symptoms of depression and anxiety.

## 6 Conclusion

Overall, the findings suggest that a combined approach of a mindfulness intervention with a mindfulness app did not provide additional benefits compared to the single interventions on the investigated variables. Furthermore, the mindfulness intervention and the mindfulness app 7Mind were similar to the active control condition (communication training) on the studied variables in the short, medium, and long term. In addition, a greater use of a mindfulness app can have adverse effects. Nevertheless, future studies should confirm these results outside the COVID-19 pandemic.

## Data availability statement

The raw data supporting the conclusions of this article will be made available by the authors, without undue reservation.

## Ethics statement

The studies involving humans were approved by Ethics Committee of the University of Jena, Germany. The studies were conducted in accordance with the local legislation and institutional requirements. The participants provided their written informed consent to participate in this study.

## Author contributions

CK: Conceptualization, Formal analysis, Funding acquisition, Investigation, Methodology, Project administration, Supervision, Writing – original draft, Writing – review & editing.
