# Supplementary material for: Long-term effects of combined mindfulness intervention and app intervention compared to single interventions during the COVID-19 pandemic: a randomized controlled trial
Source: Front Psychol. 2024 Mar 19;15:1355757. doi: 10.3389/fpsyg.2024.1355757 (PMC10985326; doi:10.3389/fpsyg.2024.1355757)
Supplement: Supplementary file 1 [file Table_1.DOCX]

| *Supplementary Material*  Supplementary Table 1: Descriptive statistics by time and group (Per protocol) | | | | | | | | | | | | | | | | |
| --- | --- | --- | --- | --- | --- | --- | --- | --- | --- | --- | --- | --- | --- | --- | --- | --- |
|  | Pre-intervention (T1) | | | | Post-intervention (T2) | | | | Follow-up I (T3) | | | | Follow-up II (T4) | | | |
|  | Mind-fulness +app | Mind-fulness | App | Control | Mind-fulness +app | Mind-fulness | App | Control | Mind-fulness +app | Mind-fulness | App | Control | Mind-fulness +app | Mind-fulness | App | Control |
| Outcome | *M(SD)* | *M(SD)* | *M(SD)* | *M(SD)* | *M(SD)* | *M(SD)* | *M(SD)* | *M(SD)* | *M(SD)* | *M(SD)* | *M(SD)* | *M(SD)* | *M(SD)* | *M(SD)* | *M(SD)* | *M(SD)* |
| Mindfulness |  |  |  |  |  |  |  |  |  |  |  |  |  |  |  |  |
| Observing | 28.49 (6.05) | 28.81 (4.53) | 28.46 (5.82) | 28.39 (5.57) | 29.58 (4.51) | 30.59 (3.94) | 28.74 (4.85) | 27.97 (5.11) | 28.90 (6.11) | 29.40 (4.69) | 31.00 (4.59) | 29.46 (5.27) | 29.18 (6.07) | 29.10 (5.26) | 30.38 (4.38) | 28.95 (5.37) |
| Describing | 25.67 (7.33) | 26.38 (6.10) | 28.14 (6.40) | 27.24 (6.52) | 27.30 (6.97) | 28.77 (6.00) | 29.71 (5.52) | 28.88 (5.88) | 26.79 (8.98) | 28.00 (6.76) | 30.44 (5.69) | 30.33 (5.72) | 27.68 (8.31) | 29.05 (6.52) | 32.67 (5.01) | 31.48 (4.63) |
| Acting awareness | 24.76 (5.10) | 22.38 (6.12) | 23.71 (5.13) | 24.02 (5.47) | 24.78 (4.54) | 24.46 (5.31) | 24.63 (5.94) | 23.82 (6.23) | 26.90 (4.61) | 24.17 (5.37) | 25.15 (6.10) | 25.42 (5.08) | 25.18 (6.40) | 24.91 (5.84) | 24.00 (6.68) | 24.86 (4.29) |
| Non-judging | 24.09 (8.03) | 24.93 (8.34) | 25.16 (8.59) | 24.74 (7.89) | 26.68 (8.15) | 27.05 (8.69) | 29.20 (7.73) | 25.68 (9.38) | 29.90 (7.59) | 29.60 (8.03) | 28.19 (9.61) | 26.13 (8.95) | 24.73 (7.27) | 29.10 (7.91) | 25.81 (9.99) | 28.00 (6.77) |
| Nonreactivity | 18.87 (4.45) | 17.79 (4.19) | 17.05 (5.46) | 18.72 (5.91) | 20.58 (3.92) | 20.56 (4.56) | 19.74 (4.70) | 20.68 (5.41) | 20.48 (4.85) | 20.43 (4.55) | 21.22 (5.03) | 21.13 (5.70) | 19.59 (3.33) | 21.81 (4.11) | 19.91 (5.49) | 21.67 (4.93) |
| Mindful characteristics | |  |  |  |  |  |  |  |  |  |  |  |  |  |  |  |
| Body awareness | 3.45 (0.55) | 3.58 (0.49) | 3.49 (0.63) | 3.57 (0.58) | 3.73 (0.57) | 3.88 (0.53) | 3.72 (0.58) | 3.69 (0.65) | 3.55 (0.72) | 3.81 (0.72) | 3.90 (0.59) | 3.55 (0.67) | 3.76 (0.59) | 3.84 (0.47) | 3.96 (0.42) | 3.60 (0.51) |
| Nonattachment | 4.12 (0.84) | 4.07 (0.94) | 3.96 (1.01) | 3.89 (0.89) | 4.30 (0.79) | 4.05 (1.00) | 4.04 (0.91) | 4.01 (0.92) | 4.35 (0.81) | 4.08 (1.07) | 4.33 (0.93) | 4.09 (0.91) | 4.14 (0.77) | 4.29 (0.82) | 3.87 (0.78) | 4.23 (0.99) |
| Emotional regulation | |  |  |  |  |  |  |  |  |  |  |  |  |  |  |  |
| Reappraisal | 3.22 (0.83) | 3.04 (0.88) | 3.01 (0.92) | 3.15 (0.94) | 3.28 (0.83) | 3.12 (0.92) | 3.22 (0.70) | 3.20 (1.01) | 3.37 (0.84) | 3.19 (0.98) | 3.55 (0.74) | 3.38 (0.91) | 3.25 (0.66) | 3.38 (0.75) | 3.43 (0.58) | 3.45 (0.74) |
| Acceptance | 3.02 (0.91) | 3.20 (1.01) | 3.02 (0.99) | 3.13 (1.00) | 3.24 (0.77) | 3.35 (0.96) | 3.31 (0.92) | 3.26 (0.92) | 3.32 (0.82) | 3.28 (0.85) | 3.40 (0.93) | 3.39 (1.08) | 3.14 (0.91) | 3.44 (0.97) | 3.25 (0.75) | 3.53 (0.88) |
| Rumination | 4.02 (0.77) | 4.07 (0.80) | 4.00 (0.74) | 3.90 (0.84) | 3.76 (0.75) | 3.85 (0.95) | 3.60 (0.84) | 3.55 (1.02) | 3.51 (0.89) | 3.64 (0.93) | 3.69 (0.97) | 3.59 (0.99) | 3.77 (0.82) | 3.68 (0.87) | 3.86 (0.83) | 3.71 (0.70) |
| Mental Health |  |  |  |  |  |  |  |  |  |  |  |  |  |  |  |  |
| Depression | 8.89 (4.48) | 9.95 (6.24) | 10.23 (5.17) | 9.17 (4.91) | 8.58 (4.64) | 8.57 (5.40) | 8.91 (4.95) | 9.79 (5.03) | 10.27 (5.61) | 8.11 (5.59) | 6.63 (4.75) | 8.38 (4.49) | 10.05 (5.39) | 7.71 (5.14) | 11.19 (5.51) | 8.05 (4.25) |
| Anxiety | 8.73 (5.22) | 9.38 (5.64) | 10.34 (4.55) | 9.39 (4.66) | 8.58 (4.39) | 8.16 (5.37) | 8.66 (4.43) | 8.76 (4.55) | 7.23 (4.93) | 7.79 (5.18) | 7.06 (3.55) | 7.81 (4.43) | 9.41 (4.45) | 7.48 (4.65) | 9.91 (4.83) | 8.16 (3.42) |
| Stress | 29.27 (6.86) | 28.86 (7.42) | 31.32 (6.80) | 29.52 (6.75) | 28.13 (6.53) | 27.97 (6.85) | 27.31 (7.51) | 27.82 (7.18) | 26.50 (8.43) | 27.58 (7.49) | 25.94 (6.80) | 25.44 (7.10) | 28.32 (7.09) | 27.43 (6.16) | 27.91 (6.92) | 27.90 (7.24) |
| Attentional abilities | |  |  |  |  |  |  |  |  |  |  |  |  |  |  |  |
| Concentration | 101.38 (11.28) | 102.31 (8.75) | 102.62 (8.05) | 104.30 (9.10) | 111.19 (11.29) | 111.63 (11.32) | 107.95 (9.40) | 109.97 (10.60) | 108.00 (6.08) | 109.25 (11.16) | 110.50 (9.68) | 109.29 (10.34) | -- | -- | -- | -- |
| Working accuracy | 101.69 (11.20) | 102.00 (11.07) | 103.90 (9.89) | 102.39 (10.00) | 109.30 (8.26) | 108.82 (9.74) | 109.62 (10.24) | 107.72 (12.02) | 110.33 (1.16) | 105.58 (6.59) | 110.25 (4.79) | 103.57 (7.23) | -- | -- | -- | -- |
| Working speed | 103.64 (11.49) | 104.29 (11.48) | 104.35 (11.55) | 106.15 (10.35) | 111.11 (11.83) | 111.63 (14.04) | 108.43 (13.04) | 111.55 (10.02) | 107.33 (8.15) | 109.92 (11.70) | 110.00 (12.06) | 110.71 (11.01) | -- | -- | -- | -- |

| **Supplementary Table 2: Results of the repeated measures MANOVA analyses (Per protocol)** | | | | | | | | | | | | | | | | | | |
| --- | --- | --- | --- | --- | --- | --- | --- | --- | --- | --- | --- | --- | --- | --- | --- | --- | --- | --- |
|  | **Pre-intervention – Post-intervention (T1 – T2)** | | | | | | **Pre-intervention – Follow-up I (T1 – T3)** | | | | | | **Pre-intervention – Follow-up II (T1 – T4)** | | | | | |
| **Outcome** | *V* | *F* | *df1* | *df2* | *p* | *η_p_^2^* | *V* | *F* | *df1* | *df2* | *p* | *η_p_^2^* | *V* | *F* | *df1* | *df2* | *p* | *η_p_^2^* |
| **Mindfulness** |  |  |  |  |  |  |  |  |  |  |  |  |  |  |  |  |  |  |
| Group effect | 0.11 | 1.13 | 15 | 426 | .329 | .04 | 0.25 | 1.85 | 15 | 312 | **.028** | .08 | 0.31 | 1.84 | 15 | 237 | **.030** | .10 |
| Time effect | 0.32 | 13.33 | 5 | 140 | **< .001** | .32 | 0.34 | 10.47 | 5 | 102 | **< .001** | .34 | 0.34 | 7.94 | 5 | 77 | **< .001** | .34 |
| Interaction effect | 0.15 | 1.46 | 15 | 426 | .119 | .05 | 0.07 | 0.52 | 15 | 312 | .927 | .03 | 0.17 | 0.96 | 15 | 237 | .496 | .06 |
| **Mindfulness characteristics** | |  |  |  |  |  |  |  |  |  |  |  |  |  |  |  |  |  |
| Group effect | 0.03 | 0.58 | 6 | 280 | .743 | .01 | 0.04 | 0.77 | 6 | 210 | .596 | .02 | 0.17 | 1.26 | 6 | 80 | .286 | .09 |
| Time effect | 0.22 | 19.00 | 2 | 139 | **< .001** | .22 | 0.10 | 5.51 | 2 | 104 | **.005** | .10 | 0.23 | 5.94 | 2 | 39 | **.006** | .23 |
| Interaction effect | 0.04 | 1.01 | 6 | 280 | .418 | .02 | 0.10 | 1.86 | 6 | 210 | .090 | .05 | 0.05 | 0.33 | 6 | 80 | .920 | .02 |
| **Emotional regulation** | |  |  |  |  |  |  |  |  |  |  |  |  |  |  |  |  |  |
| Group effect | 0.05 | 0.78 | 9 | 420 | .637 | .02 | 0.05 | 0.59 | 9 | 315 | .803 | .02 | 0.07 | 0.59 | 9 | 237 | .809 | .02 |
| Time effect | 0.22 | 13.31 | 3 | 138 | **< .001** | .22 | 0.28 | 13.19 | 3 | 103 | **< .001** | .28 | 0.16 | 4.91 | 3 | 77 | **.004** | .16 |
| Interaction effect | 0.03 | 0.48 | 9 | 420 | .886 | .01 | 0.07 | 0.86 | 9 | 315 | .563 | .02 | 0.08 | 0.76 | 9 | 237 | .653 | .03 |
| **Mental health** |  |  |  |  |  |  |  |  |  |  |  |  |  |  |  |  |  |  |
| Group effect | 0.02 | 0.24 | 9 | 423 | .989 | .01 | 0.17 | 1.38 | 9 | 207 | .199 | .06 | 0.11 | 1.01 | 9 | 237 | .433 | .04 |
| Time effect | 0.13 | 7.19 | 3 | 139 | **< .001** | .13 | 0.26 | 7.93 | 3 | 67 | **< .001** | .26 | 0.04 | 1.18 | 3 | 77 | .322 | .04 |
| Interaction effect | 0.08 | 1.31 | 9 | 423 | .232 | .03 | 0.12 | 0.92 | 9 | 207 | .509 | .04 | 0.14 | 1.25 | 9 | 237 | .263 | .05 |
| **Attentional abilities** | |  |  |  |  |  |  |  |  |  |  |  |  |  |  |  |  |  |
| Group effect | 0.07 | 0.74 | 9 | 300 | .672 | .02 | 0.22 | 0.58 | 9 | 66 | .812 | .07 | *--* | *--* | *--* | *--* | *--* | *--* |
| Time effect | 0.72 | 86.72 | 3 | 98 | **< .001** | .73 | 0.76 | 21.60 | 3 | 20 | **< .001** | .76 | *--* | *--* | *--* | *--* | *--* | *--* |
| Interaction effect | 0.05 | 0.59 | 9 | 300 | .809 | .02 | 0.29 | 0.79 | 9 | 66 | .627 | .10 | *--* | *--* | *--* | *--* | *--* | *--* |

**Supplementary Table 3: Results of the repeated measures ANOVA analyses (Per protocol)**

|  | **Pre-intervention – Post-intervention (T1 – T2)** | | | | | | | | | **Pre-intervention – Follow-up I (T1 – T3)** | | | | | | | | |
| --- | --- | --- | --- | --- | --- | --- | --- | --- | --- | --- | --- | --- | --- | --- | --- | --- | --- | --- |
|  | Group effect | | | Time effect | | | Interaction effect | | | Group effect | | | Time effect | | | Interaction effect | | |
| **Outcome** | *F* | *p* | *η_p_^2^* | *F* | *p* | *η_p_^2^* | *F* | *p* | *η_p_^2^* | *F* | *p* | *η_p_^2^* | *F* | *p* | *η_p_^2^* | *F* | *p* | *η_p_^2^* |
| **Mindfulness** |  |  |  |  |  |  |  |  |  |  |  |  |  |  |  |  |  |  |
| Observing | 0.78 | .507 | .02 | 3.05 | .083 | .02 | 2.00 | .116 | .04 | 0.69 | .563 | .02 | 2.53 | .115 | .02 | 0.33 | .807 | .01 |
| Describing | 1.26 | .292 | .03 | 20.48 | **< .001** | .13 | 0.66 | .579 | .01 | 1.92 | .131 | .05 | 15.35 | **< .001** | .13 | 0.85 | .471 | .02 |
| Acting awareness | 0.47 | .707 | .01 | 0.78 | .379 | .01 | 2.13 | .099 | .04 | 1.93 | .129 | .05 | 8.89 | **.004** | .08 | 0.06 | .982 | .00 |
| Non-judging | 0.52 | .672 | .01 | 27.49 | **< .001** | .16 | 1.36 | .259 | .03 | 0.77 | .514 | .02 | 43.82 | **< .001** | .29 | 0.52 | .670 | .01 |
| Nonreactivity | 0.83 | .481 | .02 | 52.22 | **< .001** | .27 | 1.34 | .265 | .03 | 0.33 | .804 | .01 | 23.60 | **< .001** | .18 | 0.46 | .713 | .01 |
| **Mindfulness characteristics** | | |  |  |  |  |  |  |  |  |  |  |  |  |  |  |  |  |
| Body awareness | 0.46 | .708 | .01 | 36.86 | **< .001** | .21 | 1.05 | .372 | .02 | 0.82 | .487 | .02 | 8.54 | **.004** | .08 | 3.56 | **.017** | .09 |
| Nonattachment | 0.62 | .601 | .01 | 6.02 | **.015** | .04 | 0.68 | .564 | .01 | 0.64 | .589 | .02 | 3.44 | .066 | .03 | 0.23 | .875 | .01 |
| **Emotional regulation** | | |  |  |  |  |  |  |  |  |  |  |  |  |  |  |  |  |
| Reappraisal | 0.67 | .569 | .01 | 2.29 | .133 | .02 | 0.46 | .712 | .01 | 0.92 | .435 | .03 | 7.92 | **.006** | .07 | 0.36 | .779 | .01 |
| Acceptance | 0.06 | .979 | .00 | 7.41 | **.007** | .05 | 0.13 | .945 | .00 | 0.22 | .881 | .01 | 3.95 | **.049** | .04 | .62 | .606 | .02 |
| Rumination | 0.65 | .587 | .01 | 38.85 | **< .001** | .22 | 0.85 | .467 | .02 | 0.11 | .955 | .00 | 36.74 | **< .001** | .26 | 1.00 | .396 | .03 |
| **Mental health** |  |  |  |  |  |  |  |  |  |  |  |  |  |  |  |  |  |  |
| Depression | 0.32 | .814 | .01 | 4.94 | **.028** | .03 | 0.88 | .452 | .02 | 0.48 | .696 | .02 | 11.77 | **.001** | .15 | 1.93 | .132 | .08 |
| Anxiety | 0.33 | .808 | .01 | 8.82 | **.003** | .06 | 1.22 | .305 | .03 | 0.11 | .955 | .01 | 12.05 | **< .001** | .15 | 0.47 | .706 | .02 |
| Stress | 0.14 | .934 | .00 | 21.05 | **< .001** | .13 | 2.41 | .069 | .05 | 0.47 | .703 | .02 | 23.12 | **< .001** | .25 | 0.65 | .585 | .03 |
| **Attentional abilities** | |  |  |  |  |  |  |  |  |  |  |  |  |  |  |  |  |  |
| Concentration | 0.47 | .708 | .01 | 232.70 | **< .001** | .70 | 0.94 | .427 | .03 | 0.04 | .990 | .01 | 45.12 | **< .001** | .67 | 0.55 | .651 | .07 |
| Working accuracy | 0.44 | .724 | .01 | 62.93 | **< .001** | .39 | 0.35 | .790 | .01 | 1.28 | .307 | .15 | 1.77 | .197 | .08 | 0.84 | .485 | .10 |
| Working speed | 0.26 | .858 | .01 | 78.59 | **< .001** | .44 | 0.72 | .541 | .02 | 0.09 | .965 | .01 | 48.10 | **< .001** | .69 | 1.11 | .367 | .13 |

**Supplementary Table 3 (continued): Results of the repeated measures ANOVA analyses (Per protocol)**

|  | **Pre-intervention – Follow-up II (T1 – T4)** | | | | | | | | | | | | | | | | | | | | |
| --- | --- | --- | --- | --- | --- | --- | --- | --- | --- | --- | --- | --- | --- | --- | --- | --- | --- | --- | --- | --- | --- |
|  | | Group effect | | | | | | | Time effect | | | | | | | Interaction effect | | | | | |
| **Outcome** | | *F* | | | *p* | | *η_p_^2^* | | *F* | | | *p* | | *η_p_^2^* | | *F* | | *p* | | | *η_p_^2^* |
| **Mindfulness** | |  | | |  | |  | |  | | |  | |  | |  | |  | | |  |
| Observing | | 0.25 | | | .860 | | .01 | | 0.56 | | | .459 | | .01 | | 0.32 | | .811 | | | .01 |
| Describing | | 3.45 | | | **.020** | | .11 | | 21.52 | | | **< .001** | | .21 | | 1.87 | | .141 | | | .07 |
| Acting awareness | | 0.19 | | | .904 | | .01 | | 1.66 | | | .201 | | .02 | | 0.42 | | .741 | | | .02 |
| Non-judging | | 2.35 | | | .078 | | .08 | | 13.99 | | | **< .001** | | .15 | | 0.64 | | .591 | | | .02 |
| Nonreactivity | | 0.59 | | | .625 | | .02 | | 16.67 | | | **< .001** | | .17 | | 0.79 | | .505 | | | .03 |
| **Mindfulness characteristics** | | | |  | | | |  | | |  | | | |  | | | |  | | |
| Body awareness | | 1.06 | | | .377 | | .07 | | 11.09 | | | **.002** | | .22 | | 0.50 | | .683 | | | .04 |
| Nonattachment | | 0.87 | | | .466 | | .06 | | 1.33 | | | .256 | | .03 | | 0.17 | | .918 | | | .01 |
| **Emotional regulation** | | |  | | |  | | | |  | | |  | | | |  | | |  | |
| Reappraisal | | 0.12 | | | .951 | | .00 | | 2.92 | | | .092 | | .04 | | 0.92 | | .435 | | | .03 |
| Acceptance | | 1.09 | | | .359 | | .04 | | 3.38 | | | .070 | | .04 | | 0.42 | | .738 | | | .02 |
| Rumination | | 0.34 | | | .796 | | .01 | | 12.95 | | | **< .001** | | .14 | | 0.50 | | .686 | | | .02 |
| **Mental health** | |  | | |  | |  | |  | | |  | |  | |  | |  | | |  |
| Depression | | 2.32 | | | .082 | | .08 | | 0.75 | | | .391 | | .01 | | 1.37 | | .258 | | | .05 |
| Anxiety | | 1.85 | | | .144 | | .07 | | 1.74 | | | .191 | | .02 | | 0.89 | | .451 | | | .03 |
| Stress | | 0.94 | | | .424 | | .04 | | 3.63 | | | .061 | | .04 | | 1.14 | | .337 | | | .04 |
| **Attentional abilities** | | |  | | |  | | | |  | | |  | | | |  | | |  | |
| Concentration | | -- | | | -- | | -- | | -- | | | -- | | -- | | -- | | -- | | | -- |
| Working accuracy | | -- | | | -- | | -- | | -- | | | -- | | -- | | -- | | -- | | | -- |
| Working speed | | -- | | | -- | | -- | | -- | | | -- | | -- | | -- | | -- | | | -- |
